# The Prevalence of Median Rhomboid Glossitis in Diabetic Patients: A Case-Control Study

**Published:** 2011-07-01

**Authors:** J Ghabanchi, A Andisheh Tadbir, R Darafshi, M Sadegholvad

**Affiliations:** 1 Department of Oral Medicine, Shiraz University of Medical Sciences, Shiraz, Iran; 2 Department of Oral Pathology, Shiraz University of Medical Sciences, Shiraz, Iran; 3 Department of Oral Prosthodontics, Dental School, Shiraz University of Medical Sciences, Shiraz, Iran; 4 Dentist, Shiraz, Iran

**Keywords:** Diabetes mellitus, Median rhomboid glossitis, Iran

## Abstract

**Background:**

Diabetes mellitus (DM) is one of the most common disorders of endocrine glands which has a worldwide distribution and is a risk factor for oral pathology so; the purpose of the present study was to investigate the association between median rhomboid glossitis (MRG) and DM.

**Methods:**

We examined 202 Iranian patients with DM aged 10-86 years and 261 healthy subjects aged 10-28 years and the diagnosis of MRG was made based on clinical features.

**Results:**

The examination indicated that 13 (6.43%) diabetic patients and 4 (1.53%) of control group had MRG.There was a significant difference in the prevalence of MRG, between patients and control group. MRG showed no association with other variables (age, sex, duration of DM, drugs, FBS, A1C).

**Conclusion:**

In the present study the prevalence of MRG in diabetics was much higher than that of controls

## Introduction

The term "diabetes mellitus" is used to identify a group of disorders characterized by elevated level of glucose in the blood. This elevation is as result of deficiency in insulin secretion or an increased cellular resistance to the actions of insulin, leading to a variety of metabolic abnormalities involving carbohydrates,fats and proteins[1]. In 1997, the American Association of Diabetes proposed a classification system for diabetes based on its etiology. Therefore, diabetes is currently classified as: Type 1 or juvenile diabetes and Type 2 or acquired diabetes. Type 1 diabetes appears in the first or second decade of life, it is caused by the destruction of pancreatic beta cells, which can be caused by a viral or an autoimmune process leading to blockade in the production of insulin[2]. On the other hand, type 2 diabetes is the result of an abnormality that can occur both at the molecular level of insulin and at the cellular level of insulin receptors[2]. The complications of diabetes that contribute to morbidity and mortality include microvascular diseases,macrovascular diseases, in particular cardiovascular diseases and perioral diseases. In addition, diabetes increases the risk of oral pathology including acute infections, periodontitis, and possibly premalignant and malignant lesions[3]. The most common oral manifestations in diabetic patients include xerostomia, burning and eventual erythema, and ulcerations, pharyngeal infections caused by Candida albicans, cheilitis, Lichen planus, gingival problems, periodontal problems,abscesses and marked loss of alveolar bone, although none of them is a pathognomonic lesion[4]. Diabetes is a far reaching epidemic that creates morbidity and mortality for millions of people in both developed and developing countries. Therefore, it is necessary that health care professionals become interested on the disease in order to provide an appropriate treatment to these patients in the different fields of knowledge. Median rhomboid glossitis, first described by Borcq in 1914, occurs in less than 1% of general population[5]. About 70-80% of cases are in men [6]. Its etiology is unknown, although it has been proposed that it may be derived from chronic candidiasis,or that it may be of embryological, inflammation, Staph aureus or even an immunological origin [5]. It typically presents in the posterior region of the dorsum of the tongue, at the midline, anterior to the lingual "V”, however, it sometimes appears in paramedial location [6]. It appears as a rounded or rhomboid painless plaque with well-defined margin, intense reddish or pinkish in color due to atrophy or depapillation and firm to palpation [5]. Due to complications of DM including the increase of susceptibility to many infections such as oral candidiasis, the purpose of the present study was to investigate the association between MRG and DM.

## Materials and Methods

The subjects in this study were 202 Iranian patients with DM (Type 1 and type 2) aged 10-86 years. All of them were referred from Valfajr Clinic and Oral Medicine Department of Shiraz Medical School (Shiraz,Iran). Control cases were 261 healthy subjects aged 10-82 years with no signs and symptoms of any disease who were sex and age matched. Data regarding age, sex, duration of DM, drug used, and paraclinical data including FBS, Hb A1C and 2HPP, 4PM,11AM were recorded in patients with DM.All subjects were informed about the research character and agreed to participate on the study by signing the free and informed consent form. The subjects were examined clinically by two trained examiners using artificial light, mouth mirror, gauze, etc;and the diagnosis was made based on clinical features,according to WHO guidelines. Results were analyzed with SPSS software (Version 11.5, Chicago,IL, USA), and T, Chi-Square and Fisher Exact tests were used to compare the results.

## Results

**Fig. 1:**
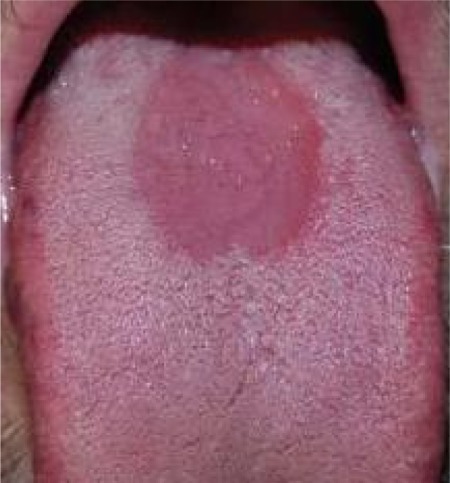
MRG in a diabetic patient

A total of 463 individuals, including 202 patients with DM (51 males and 151 females) and 261 controls (76 males and 185 females) were recruited into this study. The mean age of DM group was found to be 56.1±11.2 years and the control group 54.5± 13.4 years. In the present study, examination indicated that 13 (6.43%) of the diabetic patients and 4 (1.53%) of control group had MRG (Figure 1).

Four (7.84%) diabetic male and 5.9% (n=9) of diabetic females had MRG. This figure was 1.31% (n=1)and 1.62% (n=3) in the control group respectively.There was a significant difference in the prevalence of MRG, between patients and controls (p=0.005).MRG showed no association with other variables (age, sex, duration of DM, drugs, FBS, A1c) (Table 1 ).One of the subjects in the control group, showed kissing lesion in the palate.

**Table 1:** Data recorded in diabetic patients with and without MRG

**parameter**	**MRG+**	**MRG-**	***P* value**
Age	47.38±11.2	50.96±13.9	0.3
Duration of DM	10.54±8.2	9.2±7.9	0.5
Glibenclamid	8	88	0.3
Metformin	5	93	0.4
Glutazone	0	25	0.1
Acarbose	0	18	0.2
Insulin level	4	25	0.9
FBS	235±118	181±78	0.1
Hb A*^1C^*	9.25±0.44	8.6±1.6	0.4

## Discussion

Diabetes mellitus is one of the most common disorders of endocrine glands which has a worldwide distribution,occurring in about 1 to 2% of the word population, and it is more prevalent in well fed populations because they have better access to mostly high- calorie foods[2]. Diabetes is a risk factor for oral pathology including gingivitis, periodontitis, candidiasis,oral lichen planus, premalignant lesions like leukoplakia and oral malignancies[7][8].In this study the prevalence of MRG in diabetic patients was 6.43% in comparison to 1.53% in the control group. It occurred with significantly more prevalence in our subjects with DM than in control subjects and its prevalence was similar to another study in Iran which report the prevalence of MRG to be 7% in diabetic patients[9]. Guggenheimer et al. reported that more subjects with insulin-dependent diabetes mellitus (IDDM) than control subjects without IDDM (15.1% vs 3.0% were found to have clinical manifestations of candidiasis,including MRG, denture stomatitis and angular chelitis[10]. The prevalence of MRG was 7% in their study which was similar to the present study. Farman observed that atrophic lesion of the tongue were found in 26.4% of the diabetic patients and 91.7% of these lesions were MRG. They reported that the prevalence of MRG in diabetics was much higher than that of MRG found in previous investigations among other populations. They suggested that patients with MRG should be screened to rule out diabetes mellitus as the underlying cause[11]. Ponte et al. reported that among the inflammatory manifestations of the oral mucosa found in diabetes,glossitis deserve especial attention. Probably as a consequence of higher frequency of Candida albicans infection and microvascular changes, diabetics have a higher frequency of atrophic tongue lesions (central papillary atrophy) and of geographic tongue[12]. MRG is a recognized manifestation of chronic candidiasis[11]. It is well known that uncontrolled diabetes predisposes to a variety of superficial and systemic infections,and oral candidiasis in particular is thought to be more prevalent among these individuals. The course of infection is also more complicated in these patients group[13]. The mechanism by which diabetes predisposes to high candidial infections is not yet established. However it is widely recognized that high salivary glucose levels in diabetic patients favors yeast growth[10][14], but Quirino et al. linked this high frequency of Candida albicans infections with hyposalivation[15]. In the present study, 7.84% of diabetic males and 5.9% of diabetic females had MRG and MRG showed no association with other variables (age, sex, duration of DM drugs, FBS and A1C).Hoseinpoor showed that none of the males and 8.6% of the females had MRG[9]. Guggenheimer et al.reported that diabetic subjects with MRG were more likely to have a longer duration of IDDM and MRG was also significantly associated with older age, male gender and diabetic complications of nephropathy and retinopathy[10]. In the present study, the prevalence of MRG in diabetics was found to be much higher than that of controls.Diabetes is a prevalent disease that causes multiple co-morbidities. Oral pathologies are complications of diabetes that will bring these patients to the attention of oral health practitioners. Many of these individuals will have undiagnosed diabetes or uncontrolled diabetes,and the oral medicine physician can be critical in making the diagnosis counseling the patient in the importance of diabetes control and referring the patient to an endocrinologist for farther management.For this reason, the oral medicine physician can have a major impact on both the diagnosis and control of this common disease, there by improving the lives of individuals with diabetes.
